# Impact of sex and advancing age on left atrial volume and shape: the Framingham heart study

**DOI:** 10.1186/1532-429X-18-S1-P130

**Published:** 2016-01-27

**Authors:** Garima Arora, Philimon N Gona, Carol J Salton, David D McManus, Christopher J O'Donnell, Warren J Manning, Michael L Chuang

**Affiliations:** 1 NHLBI's Framingham Heart Study, Framingham, MA USA; 2 grid.239395.70000000090118547Cardiology, Beth Israel Deaconess Medical Center, Boston, MA USA; 3 Cardiology, University of Alabama, Birmingham, AL USA; 4 grid.266685.90000000403863207Exercise and Health Sciences, University of Massachusetts, Boston, MA USA; 5 grid.168645.80000000107420364Cardiology, University of Massachusetts Medical School, Worcester, MA USA; 6 Cardiology, VA Medical System, Boston, MA USA

## Background

Left atrial (LA) enlargement is a predictor of adverse cardiovascular disease (CVD) outcomes including atrial fibrillation, stroke, congestive heart failure, and CVD death. Recent reports suggest that LA shape is itself associated with excess burden of adverse CVD events. We sought to determine the impact of sex and age on LA volume and shape in a sample of community-dwelling adults free of clinical CVD and common clinical risk factors (apart from age) for LA enlargement.

## Methods

1794 adults from the Framingham Heart Study Offspring cohort underwent ECG-gated, breath hold cine SSFP CMR in the 2- and 4-chamber views, and a multislice, axial, T2W black-blood series encompassing the thorax. LA volume was calculated at the cardiac phase immediately before mitral valve opening using the biplane area-length formula; volume was indexed to body surface area (BSA). LA diameter was measured from the axial image with greatest anteroposterior LA dimension. We defined LA shape index (SI) as 4-chamber length divided by LA diameter (LA SI=1 would indicate a round left atrium). To minimize confounding effects of common risk factors for LA enlargement on the relationship between age, sex and LA volume and SI, we identified a referent group free of: prevalent CVD, obesity (BMI<30 kg/m2), hypertension (BP<140/90 mmHg, no antihypertensive medications), and left-sided heart murmur. Referent participants were stratified by sex and 10-year age groups. Overall sex differences were assessed using 2-sample t test; linear regression with age-group as an ordinal variable was used to test for linear trends within each sex.

## Results

Among all 1794 Offspring (aged 65 ± 9 years) who underwent CMR, the healthy referent group comprised 229 men (60 ± 8 y) and 379 women (62 ± 9 y). Men had greater raw LA volume than women (77 ± 22 vs. 63 ± 18 ml, p < 0.0001) but there were no statistically significant sex differences after BSA-indexation (38 ± 11 vs. 37 ± 11 ml/m^2^, p = 0.34). LA shape was more round in men (SI=1.73 ± 0.28) then women (SI=1.82 ± 0.31), p = 0.0003. There were no trends for increase or decrease in either raw or indexed LA volume across age-groups among men (see Table 1). Raw LA volume decreased linearly with advancing age-group among women (p for trend=0.016) but not after indexation to BSA (p for trend=0.17). LA SI decreased significantly (the LA was more rounded) with increasing age-group in each sex.

**Table 1:** Data summarized as mean ± standard deviation

Age Group	<55 y	55-64 y	65-74 y	≥75 y	P for trend
Men: N	63	106	45	14	
LA volume, ml	76 ± 20	76 ± 23	76 ± 24	69 ± 21	0.46
LA vol/BSA, ml/m2	37 ± 10	38 ± 11	39 ± 12	36 ± 11	0.94
LA shape index (SI)	1.81 ± 0.35	1.71 ± 0.23	1.70 ± 0.28	1.57 ± 0.26	0.005
Women: N	78	179	89	31	
LA volume, ml	65 ± 18	63 ± 17	62 ± 22	55 ± 16	0.016
LA vol/BSA, ml/m2	38 ± 11	37 ± 10	37 ± 14	34 ± 10	0.17
LA shape index (SI)	1.88 ± 0.27	1.83 ± 0.32	1.77 ± 0.30	1.75 ± 0.30	0.007

## Conclusions

Among adults free of clinical CVD and common clinical risk factors, other than age, for LA enlargement, we found that men have greater absolute LA volume than women, but there were no sex differences after indexation of LA volume to BSA. Indexed LA volumes did not increase or decrease with advancing age-group in either sex. Men have a more rounded LA shape than women overall, and LA shape appears to become more rounded with advancing age in both sexes. These LA shape results suggest that simple linear measurements, particularly LA diameter, may not accurately represent LA volume.

